# Atopic Dermatitis: The Fate of the Fat

**DOI:** 10.3390/ijms23042121

**Published:** 2022-02-14

**Authors:** Petra Pavel, Stefan Blunder, Verena Moosbrugger-Martinz, Peter M. Elias, Sandrine Dubrac

**Affiliations:** 1 Department of Dermatology, Venereology and Allergology, Medical University of Innsbruck, A-6020 Innsbruck, Austria; petra.pavel2@gmail.com (P.P.); stefan.blunder@i-med.ac.at (S.B.); verena.martinz@i-med.ac.at (V.M.-M.); 2 Department of Dermatology, University of California, San Francisco, CA 94115, USA; peter.elias@ucsf.edu

**Keywords:** atopic dermatitis, lipids, epidermal barrier, ceramides, eicosanoids, filaggrin, eczema

## Abstract

Atopic dermatitis (AD) is a chronic and relapsing inflammatory skin disease in which dry and itchy skin may develop into skin lesions. AD has a strong genetic component, as children from parents with AD have a two-fold increased chance of developing the disease. Genetic risk loci and epigenetic modifications reported in AD mainly locate to genes involved in the immune response and epidermal barrier function. However, AD pathogenesis cannot be fully explained by (epi)genetic factors since environmental triggers such as stress, pollution, microbiota, climate, and allergens also play a crucial role. Alterations of the epidermal barrier in AD, observed at all stages of the disease and which precede the development of overt skin inflammation, manifest as: dry skin; epidermal ultrastructural abnormalities, notably anomalies of the lamellar body cargo system; and abnormal epidermal lipid composition, including shorter fatty acid moieties in several lipid classes, such as ceramides and free fatty acids. Thus, a compelling question is whether AD is primarily a lipid disorder evolving into a chronic inflammatory disease due to genetic susceptibility loci in immunogenic genes. In this review, we focus on lipid abnormalities observed in the epidermis and blood of AD patients and evaluate their primary role in eliciting an inflammatory response.

## 1. Atopic Dermatitis

Atopic dermatitis (AD) is the most common inflammatory skin disorder worldwide, with a prevalence of 1–20% in both children and adults (http://isaac.auckland.ac.nz/index.html (accessed on 9 February 2022) [1,2,3,4,5]. It is believed to be the first step of the so-called ‘atopic march’ in which AD is followed by allergic rhinoconjunctivitis, allergic bronchial asthma, and food allergies. However, food allergies might be concomitant to AD in very young children (https://nationaleczema.org/atopic-dermatitis-and-allergies-connection/ (accessed on 9 February 2022)) [6]. AD is a complex disease whose etiology has not yet been fully deciphered due to its heterogeneity resulting from patient age, ethnicity, and lifestyle factors [7,8,9]. Moreover, although a genetic predisposition is undeniable in AD pathogenesis, the relative contribution of (epi)genetic [10,11,12,13] versus environmental factors [14,15,16] remains unknown. Heterogeneity of AD due to genetic polymorphism extends beyond filaggrin (*FLG*) loss-of-function mutations, since patients with serine peptidase inhibitor Kazal-type 5 (*SPINK5*) mutations also exhibit a severe AD-like phenotype, as do other patients with inherited disorders [17,18]. In addition, not all AD patients display an allergic systemic profile, especially patients with mild AD [19]. Thus, AD can be considered as a basket of different etiologies producing a similar phenotype. It appears more and more necessary to stratify AD according to its various endotypes to compile pathomechanisms which are specific for each endotype, which should enable a personalized prophylactic approach for many patients, but probably not all. Better knowledge of the pathomechanisms common to all endotypes would also deliver important information for designing effective pan-therapies.

### 1.1. Cellular and Molecular Abnormalities in Atopic Dermatitis

AD patients exhibit dry skin owing to impaired epidermal barrier function at all stages of the disease, which precedes or is concomitant to the development of overt skin inflammation and skin lesions. The impaired epidermal barrier is, per se, sufficient to enhance KC proliferation and synthesis of lipid, DNA, and protein in an effort to restore the barrier. This response is part of a hierarchical imperative of sustaining a fully competent barrier in a desiccating terrestrial environment and leads to mild acanthosis in non-lesional AD skin. When the skin becomes itchy, scratching potentially enhances the penetration of antigens or bacteria into the skin [20,21], and also the release of pro-inflammatory lipids, i.e., eicosanoids (see below). This likely induces a response in KCs leading to the production of inflammatory mediators such as TSLP, IL-6, IL-1, and CCL17, and the recruitment of immune cells to the skin, thereby advancing the transition from non-lesional to lesional AD [19,20,22,23]. Antigens, bacteria, and TSLP can directly activate LCs to prime Th2 cells in regional lymph nodes, which are then recruited to the skin [23,24,25,26].

The triggers involved in the development of eczematous lesions remain largely unknown, although stress, pollution, climate, microbiota, and allergens are likely involved. Non-lesional AD skin can either be of normal appearance or exhibit overt xerosis and display several morphological (acanthosis) and immunological (Th2/Th17 subclinical inflammation) abnormalities [27]. After exposure to triggers that often cannot be specified, the skin of AD patients undergoes changes leading to the development of eczematous lesions, exhibiting age-specific distribution patterns [28]. In acute AD, pruritic and eczematous skin lesions are characterized by an exacerbated Th2/Th17 immune response associated with an under-responsive innate immunity [29]. Chronic AD lesions display a complex inflammation signature (Th1/Th2/Th17/Th22/Th9) associated with keratinocyte (KC) hyperproliferation and altered terminal differentiation as well as skin superinfection, especially with *Staphylococcus* bacteria [9,19,29,30,31,32,33,34,35,36,37]. Th2-skewed adaptive and reduced innate immunity might concertedly promote the colonization of skin with *Staphylococcus aureus* in chronic lesional AD [9,38]. Interestingly, FLG breakdown products, namely urocanic acid (UCA) and pyrrolidone carboxylic acid (PCA), exert antimicrobial effects, notably against *Staphylococcus aureus* [39,40]. In line with this, epidermal models knocked down for *FLG* show increased colonization with *Staphylococcus aureus* [41]. Thus, reduced amounts of FLG in AD skin, regardless of FLG genotype, might contribute to promoting skin superinfection with *Staphylococcus* [30,42]. This potentially occurs via reduced amounts of antimicrobial peptides and FLG breakdown products rather than via alkalinization of the skin [43]. Indeed, although surface pH is increased in AD—especially in severe AD lesions—it remains in the acidic range, i.e., ≤6 [44,45,46,47].

### 1.2. Pathogenesis of Atopic Dermatitis

While it is clear that AD is a consequence of impaired epidermal barrier function associated with immune hyper-responsiveness, probably resulting from (epi)genetic modifications [10,11,13], it is not clear which abnormality—stratum corneum (SC) versus immune pathology—occurs first. The widely cited work from Kelleher et al. which purportedly showed increased transepidermal water loss (TEWL) in 2-day-old children preceding the development of AD and allergies later on [48] has been recently retracted [49]. This reappraisal of their data does not necessarily undermine the argument in favor of an initial epidermal barrier impairment in AD patients. However, an alternative readout, such as ultrastructural analysis of the epidermis and barrier recovery assay, might be more appropriate than TEWL measurements to verify epidermal barrier dysfunction in very young patients. Mechanistically, it had been hypothesized that epidermal barrier impairment enables the penetration of antigens, pollutants or bacteria into the SC, hence leading to KC and Langerhans cell (LC) activation [20]. However, although this concept is well accepted, it has never been demonstrated and further work is required to better understand disease initiation.

Reduced amounts of FLG in skin, regardless of *FLG* loss-of-function mutations, might significantly contribute to epidermal barrier impairment. Indeed, FLG deficiency provokes alterations in the lamellar body (LB) cargo system in the stratum granulosum (SG) and LB entombment, disrupted corneodesmosome structures, and corneodesmosome-derived lacunae in the SC [44,50]. In adult patients with AD, amounts of FLG are reduced, regardless of skin lesion presence or absence [51]. However, AD children display normal to increased epidermal FLG, despite a Th2 predominant skin microenvironment, questioning the contribution of FLG deficiency to AD. In light of these data, reduced FLG levels, resulting from loss-of-function mutations or from other skin microenvironment-related factors, can be envisioned as an aggravating factor rather than as a primary factor in AD pathogenesis. Moreover, the upstream signal(s) leading to FLG down-regulation in adult AD skin remain(s) ill-defined. Interestingly, *Ovo-Like Transcriptional Repressor 1* (*OVOL1*) has been identified as a susceptibility gene for AD [10]. OVOL1 is a transcription factor important for the development of epithelial tissues arising from germ cells, and is involved in the expression of skin barrier proteins, including FLG [52,53]. KCs with a weak OVOL1 pathway produce less FLG and fail to efficiently exit proliferation after extrinsic stimulation [54]. Thus, an impaired OVOL1 pathway might account for the decrease of FLG observed in adult FLG wild-type AD skin [55].

AD initiation in very young patients might result from a combination of environmental factors (e.g., climate, airborne and food allergens, pollution) and the relative immaturity of young skin (e.g., simple skin microbiota, low skin innate immunity, immature adaptive immunity) [56,57,58,59,60,61] synergizing to heighten vulnerability. This predisposition might contribute to AD onset in young children with single nucleotide polymorphisms (SNPs) in immunogenic genes [10,13,62]. Then, as the skin matures in these children, it becomes less permeable to environmental triggers, hence explaining the progressive resolution of the disease with age. Non-resolution of the disease or disease relapse in early adulthood might result from constant exposure or re-exposure, respectively, to strong environmental elicitors (e.g., pollution, stress, climate, changes in the skin microbiota) able to chronically trigger Th2 inflammation. The latter has been shown to weaken the epidermal barrier, hence perpetuating a pathogenic vicious cycle [63].

Dry skin is an important component of AD. A recent study showed that, in very young children with AD, dry skin originates from reduced amounts of natural moisturizing factors (NMFs), which leads to corneocyte stiffening [47,64]. Although FLG breakdown products constitute an important source of NMFs, reduced amounts of NMFs in the epidermis of very young patients are not related to FLG status [27,65]. These results support previous work showing that FLG genotype is not involved or has little involvement in disease initiation in very young AD patients [20,27], despite FLG deficiency inducing changes in the lamellar body cargo system and epidermal barrier ultrastructure [44,50].

The role of food allergies as an initiator of AD is still controversial [66]. Food allergies are known to exacerbate AD, but it remains unclear whether food allergies, notably to formula milk, might compromise the epidermal barrier of young AD patients, hence initiating the disease [67]. A recent study in children aged 4–7 showed that epidermal barrier impairment is more pronounced in AD patients with food allergies than in those without known food allergies; however, AD patients without food allergies still display abnormal epidermal barrier function [68].

Exposure to pollutants, allergens, specific microbes and low ambient humidity might lead to impaired epidermal barrier and dry skin via profound and sustained modification of lipid metabolism in the epidermis (https://doi.org/10.3389/fenvs.2014.00011 (accessed on 9 February 2022)) [69,70,71,72], hence contributing to AD pathogenesis [21,73]. For example, low humidity steepens the gradient of water loss across the SC, thereby placing extra stress on an already flawed epidermis. Moreover, alterations of lipid availability in the AD skin microenvironment might play a role in the antimicrobial innate immune response and influence the fate of local skin dendritic cells (DCs) [22].

## 2. Reduced Lipid Amounts in the Epidermis of Patients with Atopic Epidermis

The SC is composed of corneocytes embedded in a lamellar lipid matrix consisting of ceramides (CERs), free fatty acids (FFAs), and cholesterol (CHOL). The AD epidermis displays reduced levels of total lipids, phospholipids, and sterol esters, as well as increased amounts of FFAs and sterols when compared to an aged-matched healthy epidermis. In a mouse model of lesional AD, namely flaky tail mice, the expression of fatty acid synthase (FASN) is increased when compared to control mice [74]. Increased FFA synthesis in AD epidermis is likely a response to the increased demand for structural lipids in newly forming SC lipid matrix and cell membranes in neoproduced KCs—i.e., KCs are hyperproliferating in lesional AD—and as an energy substrate to sustain the KC proliferation [74,75]. Decreased amounts of phospholipids might reflect a decrease in sphingolipid content, specifically sphingomyelin [75]. This might be due to augmented utilization of those lipids for membrane synthesis and/or from enhanced activity of phospholipase A_2_ (PLA_2_). In line with this, the expression of secretory PLA_2_ (sPLA_2_) is enhanced in the sub-corneal layers of the AD epidermis, especially in FLG mutated patients [cytosolic PLA_2_ (cPLA_2_) is not increased in AD]. Increased hydrolysis of phospholipids leads to the liberation of FFAs, resulting in a higher amounts of pro-inflammatory bioactive lipid mediators, including arachidonic acid (AA) and other eicosanoids in AD skin [50,76,77,78,79]. Moreover, higher levels of monounsaturated FFAs have been consistently observed in the AD epidermis compared to that of healthy controls, and might contribute to epidermal barrier alteration [77,80,81,82].

Epidermal content of free CHOL increases in AD versus healthy subjects [83], and is associated with increased synthesis via 3-hydroxy-3-methyl-glutaryl-coenzyme A (HMGCoA) reductase. This might also occur as part of an attempt by the epidermis to restore the SC barrier [84]. The content of CHOL-3-sulfate is increased in AD skin [85]. However, CHOL sulfate has been shown to dose-dependently increase FLG expression in KCs via retinoid-related orphan receptor alpha (RORα [86]. The latter observation contrasts with data showing reduced amounts of FLG in AD skin, at least in adult patients with moderate to severe AD [19,87].

**Figure 1:**
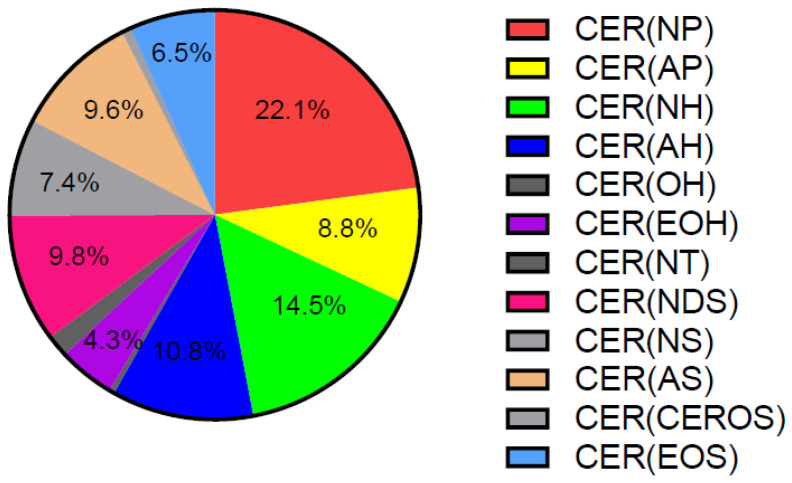
Distribution of CER classes in human healthy SC. Adapted from [93]. CER: ceramide, SC: stratum corneum.

Approximately 50% of SC lipids are CERs, and alterations in their quantity and composition cause changes in epidermal barrier properties [21,73,88]. Both total CERs, when expressed as percentage of total lipids, and ultra-long-chain CERs (C ≥ 26 carbons) are reduced in the SC of both lesional and non-lesional AD [89,90,91]. This is in line with the observation of lamellar bodies with reduced lipid content in the SG of patients with AD, leading to their partial entombment in corneocytes [50]. CERs are divided in different subclasses containing α-hydroxy fatty acid (A), ω-hydroxy fatty acid (O), non-hydroxy fatty acid (N), ester-linked non-hydroxy fatty acid (E), sphingosine (S), phytosphingosine (P), or 6-hydroxy-sphingosine moieties (H). The levels of CER [AS], CER [NS] and total C34 CERs all positively correlate with TEWL, as opposed to CER [EOP], CER [EOH], CER [NP], CER [NH], and total CER [EO] [92]. Other work showed that the SC content of CER [NS] is not significantly altered in AD [89]. CER [EOS] and CER [EOH] are reduced only in the SC of AD lesions, whereas CER [NP] and CER [NH] are additionally reduced in the SC of non-lesional AD skin [89,90]. CER [NP] is the most abundant CER class, accounting for approximately 22% of all CERs in human healthy SC (Figure 1) [93].

Decreased amounts of CERs in the SC of AD patients are not due to increased degradation via endogenous ceramidase [90,94,95]. It has been hypothesized that enrichment of the skin microbiota with ceramidase-secreting bacteria might contribute to reduced levels of CERs in the SC of both lesional and non-lesional AD skin [96]. However, *Staphylococcus aureus* does not exhibit ceramidase activity, suggesting that other bacteria would be involved [96]. While *Pseudomonas aeruginosa* secretes ceramidase, the skin of AD patients is rarely colonized with this pathogen [30,97,98,99]. To date, no bacteria colonizing the skin of patients with AD have been identified as a relevant source of ceramidase. Hence, it can be concluded that increased consumption and/or decreased synthesis of SC CERs lead to their lower levels in AD skin. Indeed, Th2 cytokines such as IL-4 have been shown to diminish CER synthesis in KCs via signaling through the STAT6 or ERK1/2 pathway [100]. However, the synthesis of CERs seems to be augmented in AD epidermis, as shown by increased immunostaining for CER synthase 3, β-glucocerebrosidase, and acid-sphingomyelinase in non-lesional and lesional AD skin when compared to healthy skin [101]. Nevertheless, increased expression of enzymes involved in CER synthesis does not necessarily translate into increased CER epidermal content. Indeed, a lack of substrates can be a significant limiting factor in CER synthesis in AD epidermis. In line with this, decreased availability of phospholipids, as evidenced in AD epidermis, might contribute to reduced amounts of CERs; however, metabolic changes leading to lower amounts of CERs in AD remain to be fully deciphered.

Beside epidermal lipids, lipids of the sebum can significantly account for lipid abnormalities in AD. Sebum is a lipid-rich matrix composed of glycerolipids (30–50%), FFAs (15–30%), CHOL (1.5–2.5%), CHOL-esters (3–6%), squalene (12–20%), and wax-esters (26–30%). Hypoplastic sebaceous glands have been reported in AD, regardless of skin lesions and associated with slow sebocyte proliferation. Moreover, reduced amounts of total lipids, squalene, and wax-esters have been observed in patients with AD, as opposed to CHOL and CHOL-esters [102,103]. Moreover, patients with AD (outside of the flare period) exhibit reduced levels of sebum CERs (C36 [NS] and [NdS] CERs), regardless of sex [104]. Alterations in the composition of sebum lipids in patients with AD might contribute to changes in skin microenvironment with potential pathophysiological consequences by affecting bacterial and fungal microbiota and skin physicochemical properties. 

Alterations in the lipid composition of the epidermis can directly hamper innate immunity in AD and, in turn, favor microbial superinfection [20,75,105]. SC lipids such as sphingosines, sphingosine derivatives, and several FFAs exert antimicrobial properties and are thus considered as antimicrobial lipids. This was demonstrated experimentally by the topical application of lipids isolated from the SC of mice fed an FFA-poor diet onto the skin of human subjects following inoculation with *Staphylococcus aureus*—the lipids showed strong antimicrobial activity [106]. This study suggests that epidermal barrier impairment, due to an FFA-poor diet, results in higher secretion of antimicrobial lipids. Interestingly, sphingosines exert antimicrobial properties at physiological doses. The SC contains approximatively 0.8 mg/cm^2^ sphingosines, and 2 μg/mL sphingosine or phytosphingosine or 8–60 µg/mL dihydrosphingosine can efficiently kill *Staphylococcus aureus* [106,107], whereas 6–12 µg/mL phytosphingosine or 6–18 µg/mL sphingosine are necessary to kill *Candida albicans* [106]. FFAs can also exert antibacterial and antifungal properties at nanomolar doses, especially long chain fatty acids (LCFAs) such as linoleic (LA, C18:2, Δ9, 12) and linolenic (C18:3, Δ9, 12, 15) acids [107,108,109]. Antimicrobial lipids exert cytotoxic effects and exhibit chemoattractant properties toward T-lymphocytes [110,111]. Indeed, lysophosphatidylcholines, which are the main epidermal phospholipids, induce T-lymphocyte chemotaxis in vitro [111]. In AD skin, lower content in LCFAs, sphingosines, and lysophosphatidylcholines might contribute to lessen innate immunity. However, the mechanisms of action of antimicrobial lipids have never been thoroughly investigated.

Permeability and antimicrobial barriers are intertwined and co-regulated [112,113]. Thus, in AD patients, changes in lipid content lead to changes in the lipid ratio in the epidermis, including the CHOL/FFA/CER ratio in the SC, which modifies the physicochemical properties of the permeability barrier. This might weaken epidermal barrier function as well as impair innate immunity, which could, in turn, create conditions favorable to the development of AD in patients with pre-existing genetic susceptibility in immunogenic genes. Interestingly, the skin of newborns and very young children contains reduced amounts of antimicrobial lipids when compared to adult skin [56,57].

## 3. Shorter Lipid Moieties in Epidermal Fatty Acids in Atopic Dermatitis

The length of FFAs and FAs in CERs of the SC matters since it affects the efficacy of the permeability barrier [21,73,91]. Ishikawa et al. reported reduced levels of longer chain (<50 total carbons) and higher levels of shorter chain species (>40 total carbons) in the CER [NS] class in the SC of AD patients at lesional sites when compared to healthy skin [89]. In addition, Janssens et al. found chain reduction in several CER classes in the SC of non-lesional AD skin [92]. Notably, they showed that CERs with a shorter chain length (C34 CERs) are increased within CER [AS], [AH], [NS], and [NH], whereas the VLC-ester-linked CER [EO] subclass is reduced in the SC of non-lesional AD skin compared to control skin [92]. Van Smeden at al. showed reduced proportions of CER species with more than 42 carbons and increased proportions of species with less than 42 carbons in the SC of lesional AD skin. In the CER [NS] and CER [AS] classes, the proportions of C34 CERs are higher in lesional AD when compared to healthy skin. Of note, an approximately 50% reduction in VLC-AcylCERs (CER [EOS], [EOP], [EOH] and [EODS]) is observed in lesional AD when compared to healthy skin [80]. These changes are more pronounced in lesional AD than in non-lesional AD but independent of *FLG* genotype [80]. In line with aforementioned data, CER species bound to the cornified envelope in the SC of lesional AD skin display shorter chain length [114]. Altered CER chain length distribution leads to aberrant lipid organization (increased hexagonal packing) and compromises epidermal barrier function in AD patients, correlating with TEWL values and disease severity but not with FLG status—CER [EOH] and CER [AS] show the strongest associations [92,115,116]. Thus, these results demonstrate, that there is no direct relationship between *FLG* loss-of-function mutations and changes in SC CER profile in AD. This is in line with recent work showing no qualitative alterations in CER classes in the skin of FLG-deficient mice [74]. It is also important to note that not only CER, but also sphingoid base levels (breakdown products of CER), as well as their molecular ratios, change in AD SC, and contribute to its altered properties [117]. 

The chain length of SC FFAs also affects barrier function [80]. Most SC FFAs are saturated and FFAs with C18, C24, and C26 are the most abundant species, with FFAs with ≤24 carbons representing approximately 30% of SC FFAs. Furthermore, FFAs with up to 36 carbons have been identified in human SC. In saturated FFAs, the proportions of VLCFAs (≤24 carbons) are strongly reduced, and shorter species (C16 in particular) are increased in AD [91]. Moreover, the proportions of C16:1 and C18:1 MUFAs are increased in AD SC at the expense of longer monounsaturated FFA species [77,80]. Total amounts of hydroxy-FFAs are decreased in AD SC and their relative distribution shows a lowered abundance of species with more than 18 carbons [80]. However, the compositions of other lipid species are also affected in AD. Berdyshev et al. found the proportions of long-chain sphingomyelins and lysophosphatidylcholines to be significantly lower in the SC of lesional AD skin compared to healthy skin [118]. Thus, in AD, all epidermal lipid species show shorter moieties, suggesting major abnormalities in lipid metabolism.

The mechanism underlying lipid chain shortening in the AD SC is not fully understood. Several studies suggest that impaired chain elongation of FAs upstream of CER assembly might be involved [101]. ELOVL1 is an endoplasmic reticulum-bound enzyme which elongates the chain of VLCFAs (from C20–22 up to C26). It has highest activity toward C22:0 acyl-CoA, and is important for the synthesis of saturated C24:0 and monounsaturated C24:1 VLCFAs, which are precursors of sphingolipids [119]. ELOVL1 immunostaining is reduced in the epidermis, including the SC, of lesional AD when compared to healthy and non-lesional AD skin [101], whereas its mRNA levels are increased in the SC of AD skin lesions [118], but decreased in the whole skin [62]. In contrast, the amounts of ELOVL6, an enzyme that elongates C12 up to C22 FFAs, are similar in AD and healthy skin, whereas its mRNA levels are reduced in lesional AD when compared to healthy and non-lesional AD skin [62,101,118]. *ELOVL3* mRNA levels (elongation of C18:0 up to C24:0) are decreased, whereas those of *ELOVL4* (elongation of C24 to C28 and longer) are increased in the SC of AD skin lesions [62,118]. *ELOVL3* mRNA levels were found to be decreased in both non-lesional and lesional skin in a pediatric AD cohort [120]. Taken together, these data do not provide a clear explanation for the shortening of lipid species via modulation of ELOVLs in AD epidermis.

Several studies have suggested that modulation of ELOVLs in AD is controlled by the skin microenvironment, especially Th2-type inflammation [101,118]. Treatment of human skin equivalents with a cocktail of Th2 cytokines (30 ng/mL of IL-4, IL-13 and IL-31) induced down-regulation of ELOVL1 mRNA levels by approximately 50% [101]. Moreover, IL-4 and IL-13 have been shown to down-regulate the expression of ELOVL6 and ELOVL3 in differentiated KCs, which is accompanied by a shift of the CER and sphingomyelin profile toward shorter species [118]. In contrast, IFN-γ, which is found in chronic AD lesions and where lipid shortening is most stringent, does not alter ELOVL expression [121]. However, the use of supraphysiological doses of cytokines in these studies precludes a definitive interpretation of the role of cytokines in lipid shortening in vivo.

FAD-dependent acyl-CoA oxidase (ACOX1) is the rate-limiting peroxisomal enzyme involved in the metabolism of VLCFAs (peroxisomal ß-oxidation). This reaction yields electrons, which are directly given to molecular oxygen (O_2_) to generate H_2_O_2_ [122,123]. VLCFAs accumulate in the blood of mice lacking ACOX1, which develop severe microvesicular steatohepatitis with increased intrahepatic levels of H_2_O_2_ [124]. Recent work has shown that ACOX1 expression and activity are increased in the epidermis of flaky tail mice (a model of lesional AD), which is associated with reduced levels of (V)LC-CERs and FFAs. Moreover, ACOX1 is increased in lesional, but not in non-lesional, skin of AD subjects when compared to healthy skin. These results suggest that an enhanced ACOX1 pathway might significantly contribute to the shortening of lipid chain length in lesional AD [74]. In this model, up-regulation of peroxisome proliferator-activated protein (PPAR) ß/δ might be the upstream signal that enhances the ACOX1 pathway [74]. Thus, impaired elongation of VLC-CERs and/or an increase in their catabolism via peroxisomal β-oxidation might both contribute to the shift toward shorter CER classes in AD epidermis.

## 4. Increased Eicosanoids in the Epidermis of Atopic Dermatitis Patients

Eicosanoids are derivatives of AA and act as bioactive lipid mediators. KCs cannot synthesize AA, thus PLA_2_-mediated hydrolysis of membrane phospholipids is probably the main source of epidermal AA. Then, AA undergoes several steps of oxidation to produce epoxyeicosatrienoic acids (EETs) via CYP450s, prostanoids (prostaglandins, prostacyclins, thromboxanes) via cyclooxygenase (COX) 1 and COX2, and hydroxyeicosatetraenoic acids (HETEs), leukotrienes, and lipoxins via lipoxygenases (LOXs). The skin contains eicosanoids in the nanogram range per milligram of tissue. Amounts of AA, 12-HETE, 15-HETE, PGE_2_, and leukotriene B4 (LTB_4_) are increased in both lesional and non-lesional AD skin when compared with control skin [125,126]. Interestingly, levels of 12-HETE and 15-HETE are also increased in the sebum of patients with non-lesional AD, although the origin of those eicosanoids is not clear [104]. In non-lesional AD, FLG status is an aggravating factor for altered eicosanoid content [50]. Accordingly, ALOX12B, a lipoxygenase-type enzyme, is increased in the epidermis of AD patients, regardless of disease stage [126]. In line with these results, increased amounts of AA and 12-HETE have been found in human epidermal equivalents (HEEs) generated with KCs isolated from non-lesional AD skin of patients with FLG loss-of-function mutations [50]. Treatment of control HEEs with AA induces up-regulation of pro-inflammatory mediators, i.e., *IL1B* and *CCL17*, whereas treatment with 12-HETE impairs the late differentiation process of KCs, including the down-regulation of hornerin (HNRN) [50]. Moreover, 12-HETE was shown to be involved in the itch reaction in mice [127]. Interestingly, LCs produce 12-HETE [128], hence potentially contributing to AD not only via their immunogenic capacity, but also through their capacity to release specific lipids in the epidermis. PGE_2_ is one of the main eicosanoids produced in KCs, and displays potent pro-inflammatory and vasodilatory as well as proliferative properties [128]. Moreover, scratching stimulates the production of PGE_2_ in the skin, in a dose-dependent manner [129]. Thus, in AD, the release of both 12-HETE and PGE_2_ upon itch might synergize to promote the transition of non-lesional to lesional skin. Increased production of those lipids in AD skin might result from compensatory mechanisms aimed at restoring the epidermal barrier after scratching. Indeed, timely production of eicosanoids such as PGE_2_ and 12-HETE has been reported in damaged tissue where they contribute to tissue repair [130]. Itch promotes the recruitment of neutrophils to skin [131] and the release of LTB_4_, one of the most potent chemoattractants for many immune cells [132,133]. The production of LTB_4_ in KCs and LCs is low [50,134,135], suggesting that most LTB_4_ in AD skin is produced in neutrophils [131]. The elevated synthesis of eicosanoid lipids from AA in AD skin is likely mediated via the up-regulation of sPLA_2_ rather than of cPLA_2_ [50,76,136,137]. Upstream signals leading to increased sPLA_2_ in AD epidermis remain undetermined, but might include cytokines (IL-1, IL-17A) and bacteria-derived molecules [138]. 

Thus, sustained alterations of eicosanoid metabolism in AD skin, probably resulting from compensatory mechanisms aimed at repairing the epidermal barrier, might eventually contribute to AD pathogenesis by promoting local inflammation via further activation of KCs and recruitment of immune cells to the skin. Itch might contribute to the transition from non-lesional to lesional skin by accentuating alterations in eicosanoid metabolism.

## 5. Contribution of Epidermal Lipids to Dendritic Cell Activation in Atopic Dermatitis

In AD epidermis, two populations of DCs cohabit, i.e., LCs and inflammatory dendritic epidermal cells (IDECs). LCs and IDECs are key players in the pathogenesis of AD, and their role in the disease has been previously reviewed [23,25,139]. IDECs are of monocytic origin and recruited from the blood via the release of inflammatory mediators from chronic lesional AD skin [140,141]. This is consistent with the observation that IDECs can be generated in vitro by the addition of IL-4 and IL-13 to the culture medium [142]. In contrast, LC activation is an early event in AD, and upstream activators include TSLP, allergens, and pathogens [24,26,143]. However, the lipid milieu has not previously been envisaged as a potential contributor to LC activation in AD. CD1a proteins are monomorphic antigen-presenting molecules, abundantly expressed on LCs, that bind self and foreign cellular lipids for display to T-cells. Increased PLA_2_ activity generates lipid neoantigens able to bind to CD1a on LCs. Increased PLA_2_ has been observed in KCs in AD skin, showing that KCs might generate lipid neoantigens able to activate LCs and prime CD1a-reactive T cells [50,144]. Interestingly, VLCFAs are inhibitory ligands of CD1a [145]. Thus, the combination of increased synthesis of lipid neoantigens via increased PLA_2_ together with the reduction of VLCFAs in the AD epidermis might contribute to the continuous activation of LCs and, potentially, to their capacity to skew T-cells toward a Th2 phenotype. Although several studies have clearly shown the ability of LCs to drive a Th2 immune response in AD, the identity of the molecules driving the Th2 phenotype is still unknown [26,143,146,147,148]. The role of epidermal lipid composition in Th2 inflammation via activation of CD1a on LCs in AD remains largely under-investigated. However, when CD1a binds to lipid antigens, activated LCs migrate to the regional lymph nodes to prime CD1a-restricted T-cells. The latter include skin-homing Th22 [149], Th2 [79,150], and Th17 [151] cells, all relevant in AD. Thus, meshing lipid and immunology research might help in identifying the phenotypic signature and metabolic pathways involved in the development of “Th2” LCs and the initial events eliciting Th2/Th17/Th22 inflammation in AD patients of all ages [27]. For a more comprehensive approach to AD pathogenesis, it would also be of interest to study how changes in the lipid microenvironment driven by abnormal KC metabolism affect the LC phenotype in AD.

## 6. Lipid Blood Parameters, Metabolic Syndrome and Obesity in AD Patients

Data on blood lipid composition in AD, notably on hyperlipidemia, are rather controversial [152,153,154,155]. Recent work showed total blood CHOL and triglycerides to be increased in a pediatric cohort of patients with AD and to positively correlate with SCORing atopic dermatitis (SCORAD). In contrast, in this cohort, HDL-CHOL inversely correlates with SCORAD, and a greater risk of AD onset in young children with high serum levels of total CHOL is emphasized [156,157]. Moreover, body mass index (BMI) is higher in AD patients aged 0–2 years and aged 12–14 years when compared to healthy aged-matched controls, and correlates with AD severity [157]. In line with these results, the likelihood of developing metabolic syndrome (OR, 1.61) and obesity (OR, 1.81) is increased in pediatric patients with AD [158,159,160]. In an adult cohort of 61,892 patients with moderate to severe AD, the association with metabolic syndrome is preserved (OR, 1.24), although less stringently than in children, but the association with obesity is lost. In this adult cohort, AD patients demonstrate dyslipidemia identical to that of the pediatric cohort [153]. However, the relationship between AD, hyperlipidemia, and metabolic syndrome remains unclear. IL-1β and Th17 cytokines present in the blood of AD patients [161,162] might alter fat tissue homoeostasis, especially in promoting local inflammation and metabolic abnormalities [163]. However, adoptive transfer of Th2 cells into immunodeficient mice fed a high-fat diet limits body weight gain, hence demonstrating a function of Th2 cells in resistance to obesity [164]. Thus, IL-1/Th17 and Th2 systemic inflammation would appear to have opposite effects in AD, unless the mouse data do not apply to human AD.

In an adult patient cohort with moderate to severe AD, the increase in blood levels of several pro-inflammatory factors associated with AD, such as matrix metalloproteases (MMPs) and tumor necrosis factor ligand superfamily member 14 (TNFSF14), might increase the risk of developing cardiovascular disease [162]. MMPs are involved in tissue remodeling in atherosclerotic plaques [165], and blood TNFSF14 is mildly associated with atherosclerosis (OR, 1.17) [166]. Moreover, the increased concentrations of various pro-inflammatory mediators such as CCL4, CCL17, CCL28, CXCL5, CXCL10, and CX3CL1 in the blood of AD patients might contribute to the formation of atherosclerotic plaques [162]. Reciprocally, adipocytes from obese patients produce cytokines such as IL-6, IL-1ß and TNF-α [163,167] which could potentially alter skin homeostasis and especially the epidermal barrier, a hallmark of AD. However, Vestergaard questions whether the association between AD and cardiovascular risk is actually linked to lifestyle, especially smoking, rather than to systemic inflammation [168]. Indeed, most studies do not take smoking habits into consideration [168], despite an association between AD and active smoking exists. However, this association is weaker in AD than in psoriasis [169,170,171]. Moreover, in a large German cohort, patients with AD did not have increased blood lipid parameters, including total CHOL and triglycerides, and did not display higher genetic risk factors for cardiovascular disease. Furthermore, no genetic overlap between AD and cardiovascular disease was found [155]. Thus, an association between AD and blood lipid disorders, obesity, metabolic syndrome and cardiovascular risk is not yet proven [153,154].

A recent study investigated the composition of eicosanoids in the blood of patients with AD. The authors found a significant decrease of lipoxin A_4_ (LXA_4_), leukotriene B_5_ (LTB_5_), docosahexaenoic acid (DHA) and maresin (MAR) and no increase of LA [126], in contrast to previous work [172]. LTB_5_ derives from 5-hydroxy-eicosapentaenoic acid (5-HEPE) and eicosapentaenoic acid (EPA), whose levels are slightly decreased in the blood of AD patients [126]. MAR derives from 14-hydroxy-docosahexaenoic acid (14-HDHA) and DHA. EPA and DHA both derive from ω3 polyunsaturated fatty acids (PUFAs), namely α-LA. Both LXA_4_ and MAR are considered as resolvins, i.e., anti-inflammatory lipid mediators [173]. Blood platelets produce 13(S),14(S)-epoxy-MAR, which is further metabolized into MAR in blood neutrophils [174,175]. The latter cells produce LXA_4_ as well [176]. Both MAR and LXA_4_ limit excessive neutrophil infiltration in tissues by increasing macrophage recruitment and promoting macrophage phagocytosis of apoptotic neutrophils [176,177,178]. Interestingly, LTB_5_ is also formed in human blood neutrophils [179]. Thus, it is likely that the α-LA–DHA axis is defective in blood neutrophils in AD patients, hence contributing to skin inflammation via the recruitment of neutrophils unable to produce sufficient amounts of resolvins, and potentially more prone to release pro-inflammatory lipid mediators such as LTB_4_ [180].

## 7. Conclusions

**Figure 2:**
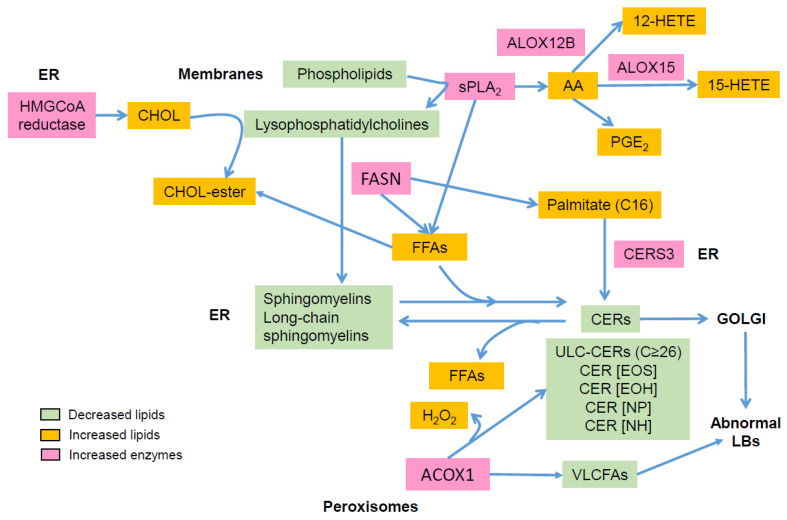
Main alterations in epidermal lipid composition in atopic dermatitis: AA: arachidonic acid; ACOX1: acyl-CoA oxidase; ALOX: lipoxygenase; CER: ceramide; CERS: ceramide synthase; CHOL, cholesterol; ER: endoplasmic reticulum; FASN: fatty acid synthase; FFA: free fatty acid; HETE: hydroxyeicosatetraenoic acid; LB: lamellar body; PG: prostaglandin; sPLA_2_: secretory phospholipase A_2_; VLCFA: very long chain fatty acid.

**Figure 3:**
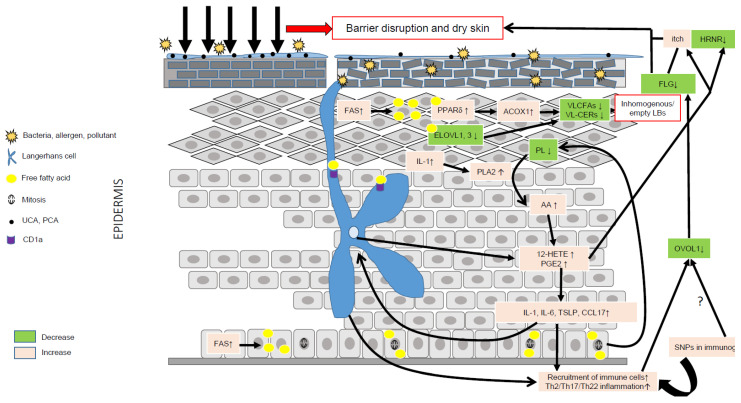
Role of lipid metabolism in AD pathogenesis. Environmental factors such as the microbiota, pollution, climate, allergens and stress can significantly alter the epidermal barrier and lead to dry skin. Compensatory mechanisms aimed at repairing the barrier include production of pro-inflammatory cytokines such as IL-1, up-regulation of FFA synthesis via FAS, increased synthesis of CHOL via HMGCoA reductase, and of proteins and DNA. IL-1 is a pleiotropic cytokine able to stimulate the hydrolysis of membrane phospholipids via PLA_2_ and the liberation of AA. AA is then metabolized into eicosanoids, such as 12-HETE and PGE_2_, together able to evoke itch, promote inflammation and reduce the expression of hornerin (HRNR), hence contributing to further impairment of the epidermal barrier. Increased synthesis of FFAs can directly activate the transcriptional activity of PPARδ, which, in turn, increases the metabolism of VLCFAs via up-regulation of ACOX1. The latter, combined with a reduction of ELOVL1 and -3, leads to reduced levels of VLC-CERs and VLC-FFAs in the epidermis, hence modifying the composition of the SC lipid matrix. VLC-CERs and VLC-FFAs are used as energetic substrates to sustain KC hyperproliferation rather than as structural lipids, hence leading to the synthesis of empty or inhomogeneous lamellar bodies (LBs). Abnormal lipid metabolism resulting in fewer antimicrobial lipids might blunt the innate immune response, hence favoring skin superinfection. Of note, the immature skin of young children contains lower amounts of antimicrobial lipids. LCs can produce 12-HETE, thereby significantly contributing to the local pro-inflammatory milieu, and, via CD1a, take up lipid neoantigens produced in activated KCs or secreted by bacteria. LCs can also sense changes in the skin microbiota, and take up allergens or directly be activated by pro-inflammatory cytokines such as TSLP. Then, activated LCs migrate to skin draining lymph nodes where they prime Th2 T cells. Th2 cytokines, such as IL-13, can down-regulate the OVOL1 pathway, potentially similar to SNPs, leading to dampened FLG synthesis and, in turn, contributing to reduced skin moisture. The secretion of abnormal LBs and the diminution of FLG associated with genetic variants (SNPs) in immunogenic genes might create a vicious cycle perpetuating AD.

AD is characterized by major aberrations in lipid metabolism (Figure 2), resulting in epidermal barrier impairment and itch and significantly contributing to inflammation via activation of KCs, LCs, and IDECs, hence emphasizing a key role of lipids in AD pathogenesis (Figure 3). Thus, it is pertinent to hypothesize that AD is primarily a disorder of lipid metabolism evolving into an inflammatory disease due to genetic susceptibility in immunogenic genes. To date, no genome-wide association studies have evidenced susceptibility loci in lipid metabolism in AD [10], confirming that abnormalities in lipid metabolism are induced by genetic susceptibility in genes unrelated to lipid metabolism and/or by environmental factors such as microbiota, pollution, climate, allergens, and stress. OVOL1 might be the missing link between FLG deficiency and abnormal lipid metabolism in AD. Indeed, OVOL1 controls the expression of FLG and a recent study revealed that lipid metabolism is the most altered metabolic pathway, with the notable up-regulation of PPARδ, in OVOL1 knock-out mice treated with imiquimod to induce psoriasis-like inflammation [181]. We have recently reported a key role of PPARδ signaling in the pathogenesis of AD and potentially of psoriasis, by up-regulating peroxisomal ß-oxidation via ACOX1, which contributes to shortening of SC lipid species, thereby initiating or perpetuating epidermal barrier impairment in AD [74]. Thus, environmental factors (e.g., climate, stress, antigens, microbiota, and pollution) or SNPs in non-immunological genes such as *OVOL1* might alone or synergistically initiate a cascade of events leading to major lipid metabolism disturbances and epidermal barrier weakening, hence creating a favorable milieu for AD development in patients with susceptibility loci in immunogenic genes. Other susceptibility loci such as FLG might contribute to aggravation of dry skin and, in turn, itch. They are probably not sufficient to initiate AD (itch per se does not lead to AD), but must synergize with other skin abnormalities to become relevant permissive factors, notably in disease progression.

## Data Availability

Not applicable.

## Abbreviations

AA: arachidonic acid; aSmase: acid-sphingomyelinase; ACOX1: acyl-CoA oxidase; AD: atopic dermatitis; ALA: alpha-linolenic acid; BMI: body mass index; CCL: C-C motif chemokine ligand; CER: ceramide; CHOL: cholesterol; COX: cyclooxygenase; CXCL: chemokine (C-X-C motif) ligand; CYP450: cytochrome P450; DC: dendritic cell; DHA: docosahexaenoic acid; EET: epoxyeicosatrienoic acids; EPA: eicosapentaenoic acid; FASN: fatty acid synthase; FFA: free fatty acid; FLG: filaggrin; 14-HDHA: 14-hydroxy-docosahexaenoic acid; HDL: high-density lipoprotein; HEE: human epidermal equivalent; HEPE: hydroxy-eicosapentaenoic acid; HETE: hydroxyeicosatetraenoic acid; HMGCoA reductase: 3-hydroxy-3-methyl-glutaryl-coenzyme A reductase; HNRN: hornerin; IDEC: inflammatory dendritic epidermal cell; KC: keratinocyte; IL: interleukin; LA: linoleic acid; LC: Langerhans cell; LOX: lipoxygenase; LT: leukotriene; LX: lipoxin; MAR: maresin; MMP: matrix metalloprotease; MUFA: monounsaturated FFA; NMF: natural moisturizing factor; OR: odd ratio; OVOL1: Ovo-Like Transcriptional Repressor 1; PCA: pyrrolidone carboxylic acid; PG: prostaglandin; sPLA_2_: secretory phospholipase A2; cPLA_2_: cytosolic phospholipase A_2_; PPAR: peroxisome proliferator-activated protein; PUFA: polyunsaturated fatty acid; RORα: retinoid-related orphan receptor alpha; SCORAD: SCORing atopic dermatitis; SNP: single nucleotide polymorphism; SPINK5: serine peptidase inhibitor Kazal-type 5; SC: stratum corneum; SG: stratum granulosum; TEWL: transepidermal water loss; TG: triglycerides; TNFSF14: tumor necrosis factor ligand superfamily member 14; TSLP: thymic stromal lymphopoietin; UCA: urocanic acid; (V)LCFA: (very) long chain fatty acid.
